# Risk and surrogate benefit for pediatric Phase I trials in oncology: A systematic review with meta-analysis

**DOI:** 10.1371/journal.pmed.1002505

**Published:** 2018-02-20

**Authors:** Marcin Waligora, Malgorzata M. Bala, Magdalena Koperny, Mateusz T. Wasylewski, Karolina Strzebonska, Rafał R. Jaeschke, Agnieszka Wozniak, Jan Piasecki, Agnieszka Sliwka, Jerzy W. Mitus, Maciej Polak, Dominika Nowis, Dean Fergusson, Jonathan Kimmelman

**Affiliations:** 1 Research Ethics in Medicine Study Group (REMEDY), Department of Philosophy and Bioethics, Jagiellonian University Medical College, Kraków, Poland; 2 Department of Hygiene and Dietetics, Chair of Epidemiology and Preventive Medicine, Jagiellonian University Medical College, Kraków, Poland; 3 Department of Public Health and Health Promotion, Regional Sanitary-Epidemiological Station in Kraków, Poland; 4 Section of Affective Disorders, Department of Psychiatry, Jagiellonian University Medical College, Kraków, Poland; 5 Agency for Health Technology Assessment and Tariff System, Warsaw, Poland; 6 Department of Rehabilitation in Internal Diseases, Jagiellonian University Medical College, Kraków, Poland; 7 Department of Surgical Oncology, Maria Skłodowska-Curie Memorial Cancer Centre and Institute of Oncology, Kraków, Poland; 8 Department of Anatomy, Jagiellonian University Medical College, Kraków, Poland; 9 Chair of Epidemiology and Population Studies, Jagiellonian University Medical College, Kraków, Poland; 10 Department of Immunology, Medical University of Warsaw, Warsaw, Poland; 11 Genomic Medicine, Medical University of Warsaw, Warsaw, Poland; 12 Laboratory of Experimental Medicine, Centre of New Technologies, University of Warsaw, Warsaw, Poland; 13 Clinical Epidemiology Program, Ottawa Hospital Research Institute, Ottawa, Canada; 14 Studies of Translation, Ethics and Medicine (STREAM), Biomedical Ethics Unit, McGill University, Montreal, Canada; Monash University, AUSTRALIA

## Abstract

**Background:**

Pediatric Phase I cancer trials are critical for establishing the safety and dosing of anti-cancer treatments in children. Their implementation, however, must contend with the rarity of many pediatric cancers and limits on allowable risk in minors. The aim of this study is to describe the risk and benefit for pediatric cancer Phase I trials.

**Methods and findings:**

Our protocol was prospectively registered in PROSPERO (CRD42015015961). We systematically searched Embase and PubMed for solid and hematological malignancy Phase I pediatric trials published between 1 January 2004 and 1 March 2015. We included pediatric cancer Phase I studies, defined as “small sample size, non‑randomized, dose escalation studies that defined the recommended dose for subsequent study of a new drug in each schedule tested.” We measured risk using grade 3, 4, and 5 (fatal) drug-related adverse events (AEs) and benefit using objective response rates. When possible, data were meta-analyzed. We identified 170 studies meeting our eligibility criteria, accounting for 4,604 patients. The pooled overall objective response rate was 10.29% (95% CI 8.33% to 12.25%), and was lower in solid tumors, 3.17% (95% CI 2.62% to 3.72%), compared with hematological malignancies, 27.90% (95% CI 20.53% to 35.27%); *p* < 0.001. The overall fatal (grade 5) AE rate was 2.09% (95% CI 1.45% to 2.72%). Across the 4,604 evaluated patients, there were 4,675 grade 3 and 4 drug-related AEs, with an average grade 3/4 AE rate per person equal to 1.32. Our study had the following limitations: trials included in our review were heterogeneous (to minimize heterogeneity, we separated types of therapy and cancer types), and we relied on published data only and encountered challenges with the quality of reporting.

**Conclusions:**

Our meta-analysis suggests that, on the whole, AE and response rates in pediatric Phase I trials are similar to those in adult Phase I trials. Our findings provide an empirical basis for the refinement and review of pediatric Phase I trials, and for communication about their risk and benefit.

## Abstract 2

Jonathan Kimmelman and colleagues report findings from their meta analysis that shows that adverse effects and response rates in phase 1 cancer clinical trials are similar for children as for adults.

## Author summary

**Why was this study done?:**

- Phase I cancer clinical trials aim to determine the safety of a new drug, and present a high risk of serious adverse events with limited prospect of therapeutic benefit.
- National and international regulations establish limits on allowable risk for research involving children.
- Little is known about the level of risk and benefit in pediatric Phase I trials in oncology.
- We designed this systematic review with meta-analysis to establish the risk and benefit associated with pediatric Phase I studies in oncology, and to compare our results with those reported for Phase I adult studies in the literature.

**What did the researchers do and find?:**

- We systematically searched for pediatric Phase I cancer studies published between 1 January 2004 and 1 March 2015.
- We identified 170 studies with 4,604 patients meeting eligibility criteria.
- The pooled overall objective response rate was 10.29%, with a response rate of 3.17% for solid tumors and 27.90% for hematological malignancies.
- The overall rate of clearly reported fatal (grade 5) adverse events was 2.09%.
- The average grade 3/4 adverse event rate per person was equal to 1.32.

**What do these findings mean?:**

- Serious (grade 3, 4, and 5) adverse event and response rates for pediatric Phase I cancer studies were similar to those reported for adult studies.
- These data help to inform the assessment and ethical evaluation of, and communication about, risk for pediatric Phase I cancer protocols.

## Introduction

Despite enormous strides in treating pediatric malignancies, childhood cancer remains the fourth leading cause of death in US children aged 1–18 years [1]. Historically, many pediatric malignancies were treated by adjusting the dosages of anti-cancer drugs that were proven effective in adults [2,3]. However, many pediatric tumors differ histologically from those of adults. Also, children’s physiology may substantially change drug pharmacokinetics and pharmacodynamics [4,5]. As a consequence, new cancer treatments must generally be validated in pediatric populations.

Phase I trials in oncology aim at establishing dose, safety, and preliminary evidence of efficacy of new cancer drugs. Participants generally have advanced cancer and have exhausted standard therapeutic options. Because Phase I trials expose patients to unproven drugs and involve a high degree of uncertainty about risk, the ethical oversight approaches have been widely debated [6–12]. In pediatric trials, participants cannot legally provide informed consent, thus adding an additional challenge to the conduct and ethical evaluation of protocols [13–22]. As with adults, Phase I trials in children present risks of serious toxicity and limited prospect of benefit, and patients are potentially exposed to levels of drug that are inactive [2,11,23,24]. Longer survival times of children can be associated with possible later side effects of cancer therapy, including secondary cancers. Several practices are designed to maximize the therapeutic prospect of Phase I pediatric cancer trials, including prior testing in adults and testing within a narrower dose range [2,4,25].

Little is known about the risk and benefit for pediatric Phase I trials and how well these trials comport with the ethical expectation that such studies offer a favorable balance of risk and therapeutic benefit. In 2005, Lee et al. suggested that the proportion of pediatric Phase I monotherapy trial participants experiencing drug-related fatalities was 0.5%, and the objective response rate was 9.6% [26]. Since 2005, major new drug classes have emerged, as have novel dosing regimens intended to improve risk/benefit balance [11,24,25,27–29]. In what follows, we used systematic review and meta-analysis to establish the risk/benefit balance for contemporary pediatric Phase I cancer studies and to appraise the value of practices aimed at improving the risk/benefit balance of Phase I studies in oncology.

## Methods

Our protocol was prospectively registered in PROSPERO (CRD42015015961) [30]. We followed PRISMA guidelines (S1 PRISMA Checklist).

### Search strategy

We systematically searched Embase and PubMed for articles and abstracts published between 1 January 2004 and 1 March 2015, using strategies that included key words and suggested MeSH and Emtree entry terms, their synonyms, and closely related words. Searches were not limited by language. The starting date of our search period was determined by the timing of the last study to our knowledge presenting data on the efficiency of pediatric Phase I trials in oncology [26]. The full search strategies were checked using the Canadian Agency for Drugs and Technologies in Health peer-review checklist; our literature search strategies and a flow diagram are presented in S1 Table and S1 Fig.

### Study selection and eligibility criteria

We included pediatric cancer Phase I studies, defined as “small sample size, non-randomized, dose escalation studies that defined the recommended dose for subsequent study of a new drug in each schedule tested” [31], as well as Phase I/II reports containing results of Phase I studies provided separately. We defined “minors” as individuals below the age of 21 years. Inclusion criteria were as follows: (1) all or most participants (over 50%) were less than 21 years old and the study was indicated as pediatric or results for pediatric participants were reported separately; (2) any malignancy (e.g., solid or hematological); and (3) assessment of chemotherapy (cytotoxic drugs) and/or targeted therapy (targeted therapy was defined as monoclonal antibodies or small molecules or antibody drug conjugates [32]). We excluded reports for studies involving (1) topical only or regionally delivered drugs (i.e., delivered directly to the tumor without any systemic effects or minimal systemic effects); (2) only the pharmacokinetics and/or pharmacodynamics of a tested treatment; (3) nonpharmacological modalities (e.g., surgery, radiotherapy, gene therapy, stem cell therapy, or any of these combined with pharmacological therapies); or (4) supportive care without anticancer agents or with other interventions not falling under targeted therapy, chemotherapy, or combined therapy categories (such as antiviral agents or nonspecific immunotherapy). All inclusion and exclusion criteria were defined prospectively in the protocol [30]. They are also listed in S2 Table.

### Data extraction

We created and piloted an extraction form, and on the basis of the pilot we refined the form and prepared the final version. Data were extracted from each publication independently by 2 reviewers (MW, MMB, MK, RRJ, AW, JP, AS, JWM, KS, MTW). All reviewers received training prior to extraction. Disagreements were resolved by discussion, and when necessary a third person, an arbiter, was involved (MMB, DN). An experienced methodologist and experienced experimental oncologist had supervisory roles (MMB, DN). In the case of duplicate publications for the same study, the results from full publication and, if possible, the most recent version were used in the extraction. Data were extracted using Google Forms. From each study, we extracted data related to study design, funding, reason for stopping the trial, patient characteristics, intervention, outcomes, and the timing of pediatric testing relative to adult testing. Because Phase I cancer studies do not generally have comparator arms or measure survival endpoints, we used objective response rate and the number of patients receiving recommended dose as proxies for therapeutic benefit [33–37].

### Data synthesis and analysis

We defined objective response rate as the proportion of participants with partial or complete response as defined by authors of the included studies; for hematological malignancies, we considered any of the various methods of measuring response (e.g., cytogenetic, molecular, or flow criteria) as acceptable. For acute leukemias, we did not count partial responses in our assessment of objective response, since anything short of complete response is not considered a benefit for these malignancies. Toxicity and adverse events (AEs) (grade 3, 4, or 5 drug-related events) were measured as defined by the Common Toxicity Criteria version 2.0 and revised versions (Common Terminology Criteria for Adverse Events version 3.0 and version 4.0).

Because large differences were observed in response rate between solid tumors and hematological malignancies, our meta-analysis was also stratified by type of cancer. For 9 studies that included both solid tumors and hematological malignancies, patients were separated for response analysis.

Pooled response rate and fatal (grade 5) AE rate were calculated within each stratum when more than 1 study provided data using meta-analytic methods. Modeling with random effects and the restricted maximum likelihood (REML) estimator was used to account for between-study heterogeneity. *I*^2^ statistics were calculated to provide a measure of the proportion of overall variation attributable to between-study heterogeneity. Differences in response rate and grade 5 AE rate between categories of type of therapy, number of drugs, and number of types of malignancies were assessed using the *Q* test for heterogeneity in meta-regression. Pooled response and grade 5 AE rate were calculated for categories of publication year (2004–2006, 2007–2009, 2010–2012, and 2013–2015) to assess changes over time. *p*-Values for trend in response and grade 5 AE rate between 2004 and 2015 were obtained from meta-regression. Meta-analysis was conducted using the metafor package (R version 3.2.3); *p* < 0.05 was considered statistically significant.

The average number of grade 3/4 AEs per person with 95% confidence interval was estimated using a Poisson regression model. In cases where a fatal event was not clearly described as treatment-related, we excluded it from our estimations of grade 5 AE rate. In order to compare risk and benefits, we analyzed a cohort of studies where both drug-related deaths (grade 5 AEs) and response were clearly reported.

## Results

### Characteristics of the trials

Our search identified a total of 7,061 citations for review. A total of 170 unique studies met full eligibility for extraction. Our sample included 74 studies of targeted drugs (43.53%), 72 studies testing classic chemotherapy (42.35%), and 24 studies testing a combination of the two (combined therapy) (14.12%). A full list of drugs tested is shown in S3 Table.

Table 1 summarizes the characteristics of the studies in our sample. The vast majority reported Phase I trials only (155 trials, 91.18%), and 15 studies reported the results of Phase I and Phase II trials (8.82%). According to references provided by the authors, most of the pediatric studies were initiated following completion of the corresponding studies in adults (111 studies, 65.29% of all trials). However, 57 studies (33.53%) did not report adult studies as having been completed. One hundred twenty-eight studies (75.29%) included only patients with solid tumors, and 33 only with hematological malignancies (19.41%). The vast majority of the studies, 144 (84.71%), used conservative dosing strategies, where the initial dose increase was <100%; 4 (2.35%) trials used aggressive dosing designs, where at least the first 2 doses increased by 100%; and another 4 trials used a “modified Fibonacci” dosing strategy (defined as a dose increased by 100% then 66% and 50%). The majority of the studies (74.12%) recommended Phase II trials, and 6 (3.53%) recommended against further testing. The majority of corresponding authors were affiliated with North American institutions (81.18%).

**Table 1:** Characteristics of studies.

Characteristic	Category	Type of therapy			Total
		Chemotherapy	Targeted therapy	Combined therapy	
**All studies**		72 (42.35)	74 (43.53)	24 (14.12)	170 (100)
**Study definition**	**Phase I**	66 (91.67)	67 (90.54)	22 (91.67)	155 (91.18)
	**Phase I/II**	6 (8.33)	7 (9.46)	2 (8.33)	15 (8.82)
**Type of tumor**	**Solid tumor**	55 (76.39)	56 (75.68)	17 (70.83)	128 (75.29)
	**Hematological**	13 (18.06)	13 (17.57)	7 (29.17)	33 (19.41)
	**Both**	4 (5.56)	5 (6.76)	0 (0)	9 (5.29)
**Number of types of malignancies**	**1 type**	8 (11.11)	11 (14.86)	2 (8.33)	21 (12.35)
	**2 or 3 types**	13 (18.06)	9 (12.16)	3 (12.50)	25 (14.71)
	**4 types**	5 (6.94)	6 (8.11)	1 (4.17)	12 (7.06)
	**More than 4**	45 (62.50)	46 (62.16)	16 (66.67)	107 (62.94)
	**Not reported**	1 (1.39)	2 (2.70)	2 (8.33)	5 (2.94)
**Initiated after study in adults**	**Ongoing study in adults**	0 (0)	1 (1.35)	0 (0)	1 (0.59)
	**After adult Phase I**	50 (69.44)	52 (70.27)	9 (37.50)	111 (65.29)
	**Unclear**	4 (5.56)	6 (8.11)	6 (25.00)	16 (9.41)
	**Not reported**	17 (23.61)	14 (18.92)	9 (37.50)	40 (23.53)
	**No study in adults**	1 (1.39)	1 (1.35)	0 (0)	2 (1.18)
**Dosing strategy**	**Aggressive**	0 (0)	4 (5.41)	0 (0)	4 (2.35)
	**Conservative**	71 (98.61)	56 (75.68)	17 (70.83)	144 (84.71)
	**Modified Fibonacci**	0 (0)	3 (4.05)	1 (4.17)	4 (2.35)
	**Unclear**	1 (1.39)	11 (14.86)	4 (16.67)	16 (9.41)
	**Not reported**	0 (0)	0 (0)	2 (8.33)	2 (1.18)
**Phase II was recommended**	**Yes**	62 (86.11)	47 (63.51)	17 (70.83)	126 (74.12)
	**No**	4 (5.56)	2 (2.70)	0 (0)	6 (3.53)
	**Unclear or not reported**	6 (8.33)	25 (33.78)	7 (29.17)	38 (22.35)
**Number of drugs**	**1 drug**	33 (45.83)	68 (91.89)	0 (0)	101 (59.41)
	**2 or more drugs**	39 (54.17)	6 (8.11)	24 (100)	69 (40.59)
**Funding**	**Private for profit**	5 (6.94)	4 (5.41)	1 (4.17)	10 (5.88)
	**Private not for profit**	2 (2.78)	0 (0)	1 (4.17)	3 (1.76)
	**Public**	15 (20.83)	25 (33.78)	6 (25.00)	46 (27.06)
	**Mixed**	34 (47.22)	28 (37.84)	10 (41.67)	72 (42.35)
	**Not funded**	1 (1.39)	0 (0)	0 (0)	1 (0.59)
	**Not reported**	15 (20.83)	16 (22.97)	6 (25.00)	38 (22.35)
**Affiliation of the corresponding author**	**Australia**	1 (1.39)	2 (2.70)	0 (0)	3 (1.76)
	**North America**	56 (77.78)	60 (81.08)	22 (91.67)	138 (81.18)
	**Europe**	10 (13.89)	11 (14.86)	0 (0)	21 (12.35)
	**Asia**	0 (0)	0 (0)	1 (4.17)	1 (0.59)
	**Not reported**	5 (6.94)	1 (1.35)	1 (4.17)	7 (4.12)

Data given as number of studies (percent).

### Characteristics of the patients

Baseline characteristics of the 4,604 enrolled patients are provided in Table 2. In 139 studies the median age of participants was below 21 years, and in the remaining 31 studies median age was not reported. In all studies included, pediatric participants were the majority. Patients’ performance status at baseline was difficult to assess as only 32 studies reported these data, and the studies used 3 different scales, depending on the age of enrolled patients (Karnofsky, Lansky, or WHO/Zubrod scale).

**Table 2:** Characteristics of patients.

Characteristic	Category	Type of therapy			
		Chemotherapy	Targeted therapy	Combined therapy	All interventions
**Enrolled patients, *n***		1,757	2,264	583	4,604
**Evaluated patients, *n* (%)**		1,615 (91.92)	2,028 (89.58)	543 (93.14)	4,186 (90.92)
Male, *n* (%)*		844 (48.04)	997 (44.04)	291 (49.91)	2,132 (46.31)
**Median age of participants, *n* of studies**	**<7 years**	2	3	0	5
	**7.0–13.9 years**	51	47	15	113
	**14.0–20.9 years**	6	13	2	21
	**≥21 years**	0	0	0	0
	**Not reported as median**	13	11	7	31
Mean percentage of patients receiving dose**	**Below recommended**	35.4	35.7	28.3	39.1
	**Recommended**	33.8	40.4	44.4	32.2

*Gender was not reported in 12 chemotherapy, 17 targeted therapy, and 4 combination therapy studies.

**Calculated as a mean of percentage of patients given recommended dose or dose below recommended dose in individual studies, weighted by the size of the study (if such data were reported).

### Surrogate clinical benefit

We defined objective response as the surrogate clinical benefit because objective response—the main read-out of treatment response used in Phase I trials—does not always predict improved survival. Objective response rates were reported in 167 of the 170 trials. There were 406 objective responses reported among 4,349 patients enrolled in the 167 trials (Table 3). The pooled overall response rate across all malignancies was 10.29% (95% CI 8.33% to 12.25%; *I*^2^ = 74.49%; Tau^2^ [estimate of between-study variance] = 0.0007). The response rate for solid tumors among 3,569 patients was 3.17% (95% CI 2.62% to 3.72%), while the response rate for hematological malignancies among 780 patients was significantly higher: 27.90% (95% CI 20.53% to 35.27%); *p* < 0.001. Response rates varied according to the type of therapy used, significantly so in solid tumors (*p* = 0.0045), while in case of hematological malignancies this relation was at the limit of statistical significance (*p* = 0.1047). Higher response rates were observed in combined therapy trials: 44.12% (95% CI 26.30% to 61.94%) for hematological malignancies and 6.44% (95% CI 3.82% to 9.05%) for solid tumors. Response rates were similar for solid tumors tested with classical chemotherapy (6.39%; 95% CI 4.60% to 8.17%) and combined therapy (6.44%; 3.82% to 9.05%).

**Table 3:** Comparison of objective response rate and toxicity in therapy subgroups by type of malignancy.

Outcome	Type of malignancy	Measure	Type of therapy			
			Chemotherapy	Targeted therapy	Combined therapy	All interventions
**Objective responses (167 studies)**	**Solid tumors**	**Number of studies**	56	58	15	129
		**Response rate (95% CI)**	6.39% (4.60–8.17)	2.52% (1.82–3.22)	6.44% (3.82–9.05)	3.17% (2.62–3.72)
		***p*-Value**	0.0045*			—
	**Hematological malignancies**	**Number of studies**	15	16	7	38
		**Response rate (95% CI)**	26.18% (15.17–37.18)	22.80% (11.51–34.08)	44.12% (26.30–61.94)	27.90% (20.53–35.27)
		***p*-Value**	0.1047*			<0.001**
**Fatal (grade 5) AEs (70 studies)**	**Solid tumors**	**Number of studies**	18	27	2	47
		**Grade 5 AE rate (95% CI)**	1.09% (0.15–2.03)	1.70% (0.82–2.59)	1.68% (0.10–4.32)	1.85% (1.14–2.56)
		***p*-Value**	0.835*			-
	**Hematological malignancies**	**Number of studies**	10	9	4	23
		**Grade 5 AE rate (95% CI)**	3.70% (1.01–6.39)	3.16% (0.69–5.63)	6.33% (1.15–11.51)	4.04% (2.18–5.89)
		***p*-Value**	0.28*			0.14**
**Grade 3/4 AEs (129 studies)**	**Solid tumors**	**Number of studies**	41	51	11	103
		**Mean number of grade 3/4 AEs per person (95% CI)**	2.01 (1.93–2.10)	0.79 (0.74–0.85)	1.76 (1.61–1.93)	1.34 (1.22–1.47)
		***p*-Value**	<0.001***			—
	**Hematological malignancies**	**Number of studies**	11	9	6	26
		**Mean number of grade 3/4 AEs per person (95% CI)**	0.98 (0.87–1.10)	0.75 (0.65–0.87)	2.75 (2.46–3.07)	1.22 (1.12–1.31)
		***p*-Value**	<0.001***			0.01***

*p-Value from *Q* test for heterogeneity comparing response rate and grade 5 AE rate between type-of-therapy groups.

**p-Value from *Q* test for heterogeneity comparing response rate and grade 5 AE rate between types of tumor.

***p-Value from Poisson regression.

AE, adverse event.

We also found significant differences in response rate related to the number of drugs used per study, regardless of the type of therapy (S4 Table). The response rate was higher in all studies where 2 or more drugs were tested in comparison to single-drug studies (Tables 1 and S3). The highest relative difference between response rates was identified in solid tumors. For cancers treated with 1 drug, the response rate was 2.49% (95% CI 1.88% to 3.11%), while for cancers treated with 2 or more drugs, it was 10.54% (95% CI 7.61% to 13.46%); *p* < 0.001. Another significant difference between responses was related to the number of types of malignancies included in a study. The response rate was much higher in all interventions where 3 or fewer types of cancers were treated in comparison to the studies with 4 or more types of malignancies. The highest relative difference between responses was again identified in solid tumors. When 3 or fewer types of malignancies were included in a study, response rate was 15.01% (95% CI 6.70% to 23.32%). When 4 or more different malignancies were included in a study, response rate was 2.85% (95% CI 2.28% to 3.42%); *p* < 0.001.

**Fig 1:**
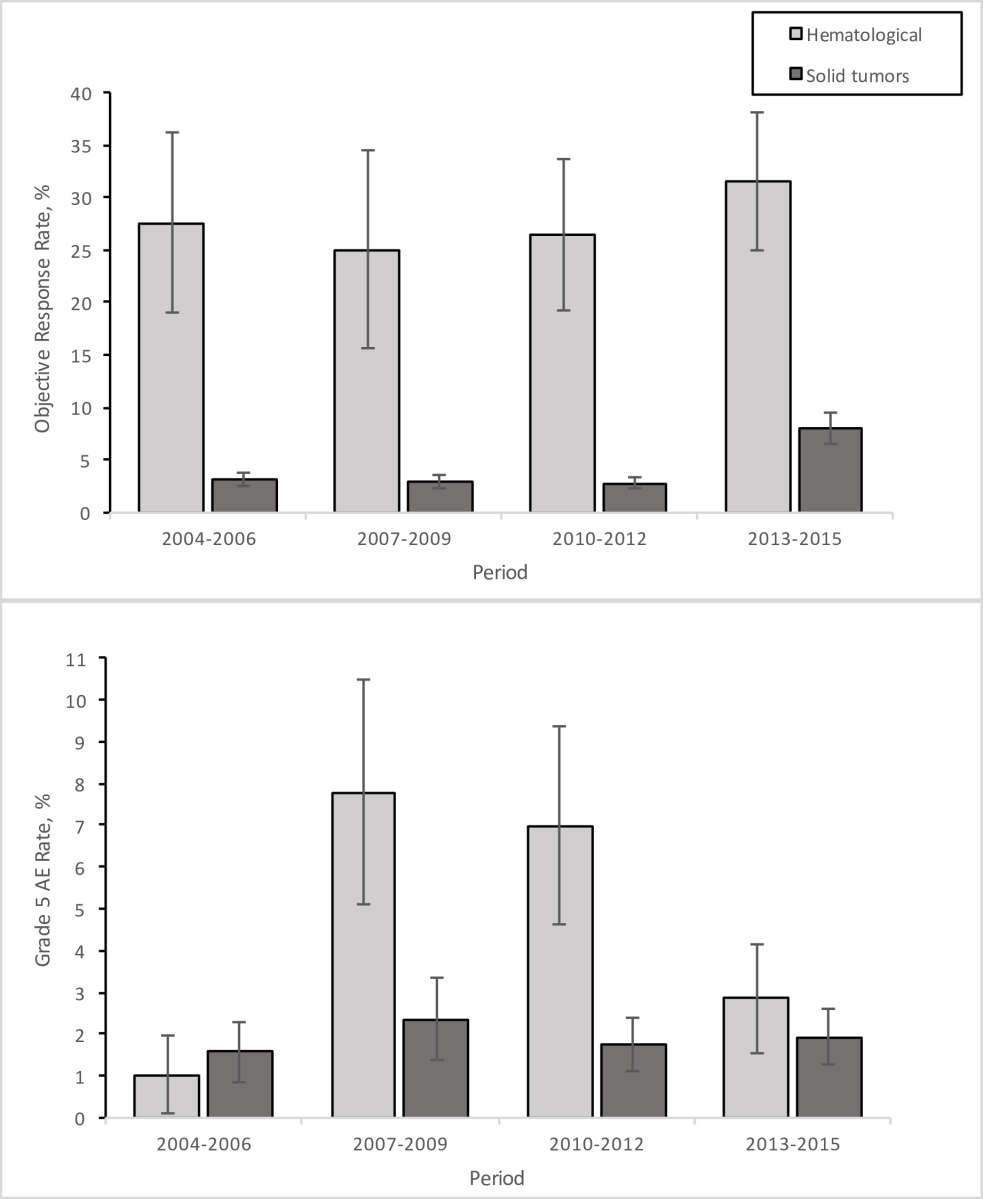
Time trends in response and treatment-related toxicity. Response and fatal (grade 5) adverse event (AE) rates were calculated for categories of publication year (2004–2006, 2007–2009, 2010–2012, and 2013–2015) to assess changes over time. Error bars indicate standard error.

We did not find significant linear time trends in objective response rates (*p* = 0.25 for solid tumors, *p* = 0.64 for hematological malignancies) (Fig 1). Table 3 shows details of response rates and fatal (grade 5) AE rates in different therapy subgroups.

### Adverse events

A total of 70 of the 170 trials reported fatal (grade 5) AEs. We observed 37 grade 5 AEs clearly reported among 1,838 patients (Table 3), suggesting an overall grade 5 AE rate of 2.09% (95% CI 1.45% to 2.72%; *I*^2^ = 0.0%; Tau^2^ = 0). This included 14 patients with solid tumors (1.85%; 95% CI 1.14% to 2.56%) and 23 patients with hematological malignancies (4.04%; 95% CI 2.18% to 5.89%). Differences in AE rates by type of therapy were not statistically significant. Grade 3/4 AEs were reported in 129 studies among 3,547 patients, with an average rate per person of 1.32 (95% CI 1.28 to 1.36). This included on average 1.34 (95% CI 1.22 to 1.47) grade 3/4 AEs per person with solid tumors and 1.22 (95% CI 1.12 to 1.31) grade 3/4 AEs per person with hematological malignancies. The highest average grade 3/4 AE rate per person was identified in patients with hematological malignancies tested with combined therapies: 2.75 (95% CI 2.46 to 3.07). We did not find significant linear time trends in fatal (grade 5) AEs (*p* = 0.9 for solid, *p* = 0.7 for hematological) (Fig 1). However, there was a significant difference in grade 5 AE rate (*p* = 0.02) for hematological malignancies between 2004–2006 (1.03%; 95% CI 0.10% to 2.87%) and 2007–2009 (7.79%; 95% CI 2.52% to 13.06%).

### Direct comparison of risk and benefit

For direct risk and benefit evaluation, we identified a cohort of 66 studies out of the 170 where both objective responses and grade 5 AEs were reported (S5 Table). For sensitivity analysis, we calculated response rates in the subgroup of 66 studies and compared them with response rates in the rest of the 101 studies where objective responses were reported. There were no statistical differences between these 2 groups in the case of solid tumors (2.97% versus 3.31%, *p* = 0.54) and hematological tumors (26.74% versus 29.42%, *p* = 0.81). We also calculated the grade 5 AE rates in the subgroup of 66 studies, and the majority of the results were almost identical as in the 70 studies where grade 5 AEs were reported. We found that higher response rates were associated with higher grade 5 AE rates in hematological malignancies. We did not find this relationship in solid tumors.

## Discussion

Our findings suggest that, on average, 1 in 10 children who enroll in pediatric Phase I trials experience objective response, while 1 in 50 die from drug-related AEs.

Because pediatric Phase I cancer trials enroll populations that lack competence to provide informed consent, these trials are generally pursued in a manner that maximizes their therapeutic prospect and reduces their risk. For example, they are generally pursued only after adult trials have clarified toxicity and appropriate dosing, and they generally test a narrower dose range. Despite this, our findings suggest that pediatric Phase I studies have similar drug-related serious (grade 3, 4, and 5) AE and response rates as adult studies. In S6 Table we compare our results with 6 similar reviews of adult Phase I cancer trials and 1 review of trials in pediatric populations. Despite the differences in methods applied in these studies, the pooled overall response rate for all types of cancers (solid and hematological) in our study was similar to that presented in meta-research with adults (10.6%) [38] and much higher than that in another study (2.95%) [39]. The pediatric response rate in our study for solid tumors, 3.17% (95% CI 2.62% to 3.72%), was slightly lower than that in adult solid tumor trials (3.8%) [40] and much lower than results presented in a smaller study (7.2%) [41]. We should further note that our aggregate objective response estimate for pediatric studies does not appear to have been driven by a small number of Phase I trials with large dose expansion cohorts. Only 44 trials involved dose expansion cohorts. Response rates for these trials did not differ from those not having dose expansion cohorts (*p* = 0.10), nor did we observe an obvious relationship between higher response rate and higher number of patients in expansion cohorts (Spearman’s rank correlation coefficient *R* = −0.08, *p* = 0.7; see S2 Fig).

The overall death rate calculated in our systematic review was also higher in comparison with non-pediatric trials, though the size of the difference may be caused by differences in the calculation method [38,40] (S6 Table). Despite an evolution in new treatments and study methods, we did not find linear time trends in risk and benefit across the time period of our analysis.

The number of patients receiving doses recommended for subsequent testing can be interpreted as another proxy of therapeutic value for Phase I trials [38], though it should be noted that, on the one hand, a minority of drugs completing Phase I studies are ultimately proven safe and effective, while, on the other hand, doses lower than those recommended can still be therapeutic (if suboptimal). Overall, 32% of the patients received the recommended dose and 39% received doses below that recommended (weighted mean). Designs intended to increase the number of patients receiving the recommended dose [2,28,42–45] were uncommon.

We found a significantly higher overall response rate in hematological malignancies than in solid tumors. This likely reflects different criteria used to assess response, differences in the biology of these malignancies, and that the former typically enroll a more homogeneous set of indications. The response rate was also higher in all interventions where 2 or more drugs were tested in comparison to the single-drug studies. The response rate was higher in all interventions where 3 or fewer types of malignancies were treated in comparison to the studies with 4 or more malignancies. This possibly indicates that studies where patients with a wider variety of malignancies are enrolled are based on a weaker research hypothesis regarding the efficacy of the tested agent against the specific malignancy. The average grade 3/4 AE rate per person was 1.32, which means that the typical patient was exposed to at least 1 major side effect of a therapy.

Our findings should be interpreted in light of the following limitations. First, the trials analyzed in our review were very heterogeneous. We used broad inclusion criteria to summarize the global response rate and risk. To reduce heterogeneity, we separated therapy types (chemotherapy, targeted agents, and combined therapies) and cancer types (solid tumors and hematological malignancies). We also explored this heterogeneity using meta-regression. Second, we relied only on published data and on the quality of reporting. Many current studies illustrate discrepancies between clinical trial registry records and published articles [46–49]. Moreover, we identified serious issues with reporting in our set of 170 analyzed trials. For instance, the poor quality of outcome reporting did not allow us to meta-analyze grade 3/4 AEs, and we were able to pool only the average number of grade 3/4 AEs per patient. Third, there was no explicit information about treatment-related deaths (grade 5 AEs) in 58.82% of studies—a figure that is surprising given the goal of Phase I trials. The low number of clearly reported treatment-related grade 5 AEs is an important limitation of our data synthesis. Fourth, response rates were used as a surrogate for benefit in our study. On the one hand, response rates could be a sensitive measure of benefit in the context of pediatric malignancies, given their rapid progression. On the other hand, the relationship between response rates and patient-centered outcomes like quality of life or survival is variable [33–37]. Moreover, eventual drug approvals are usually based on survival data from randomized controlled trials, and only about 6.7% to 9.6% of drugs tested in oncology will eventually be registered [50,51]. Better measures of benefit, like progression-free or overall survival, are typically not available in Phase I trials. Our measure of safety did not consider potential downstream effects, like secondary malignancies.

In adult Phase I cancer research, there is a lively debate as to whether access to treatments through trials is therapeutic [6–9,11,24,52]. This debate has particular significance for pediatric trials, since national and international policies generally require that interventions in trials presenting greater than minor increase over minimal risk must “hold out the prospect of direct benefit for the individual subject” and that “the relation of the anticipated benefit to the risk is at least as favorable to the subjects as that presented by available alternative approaches” [53]. Although experimental treatments in Phase I studies that deliver active drug doses clearly meet the first condition, the favorability of risk against benefit in comparison with alternative treatment options is subject to interpretation and may vary depending on the trial. Our data, coupled with careful ethical analysis, provide an empirical basis for further discussions about the therapeutic status of Phase I trials in children. In particular, they provide evidence for refining risk/benefit balance in Phase I trials and identifying those studies that present greater challenges for meeting standards of acceptable risk in children. They also provide a basis for clearer communications about risk and benefit to patients and their guardians.

## Supporting information

S1 PRISMA Checklist (DOCX)

S1 Fig PRISMA flow diagram. (TIF)

S2 Fig Relation between objective response rate and percentage of patients in dose expansion cohort. (TIF)

S1 Table Search strategy. (DOCX)

S2 Table Inclusion and exclusion criteria. (DOCX)

S3 Table Drugs and drug combinations used in included studies. (DOCX)

S4 Table Objective response rate and toxicity assessed in subgroups. (DOCX)

S5 Table Direct comparison of risk and benefit. (DOCX)

S6 Table Results of previous reviews. (DOCX)

## Abbreviation

AE: adverse event
