# Supplementary figures and images for: Risk and surrogate benefit for pediatric Phase I trials in oncology: A systematic review with meta-analysis
Source: PLoS Med. 2018 Feb 20;15(2):e1002505. doi: 10.1371/journal.pmed.1002505 (PMC5819765; doi:10.1371/journal.pmed.1002505)

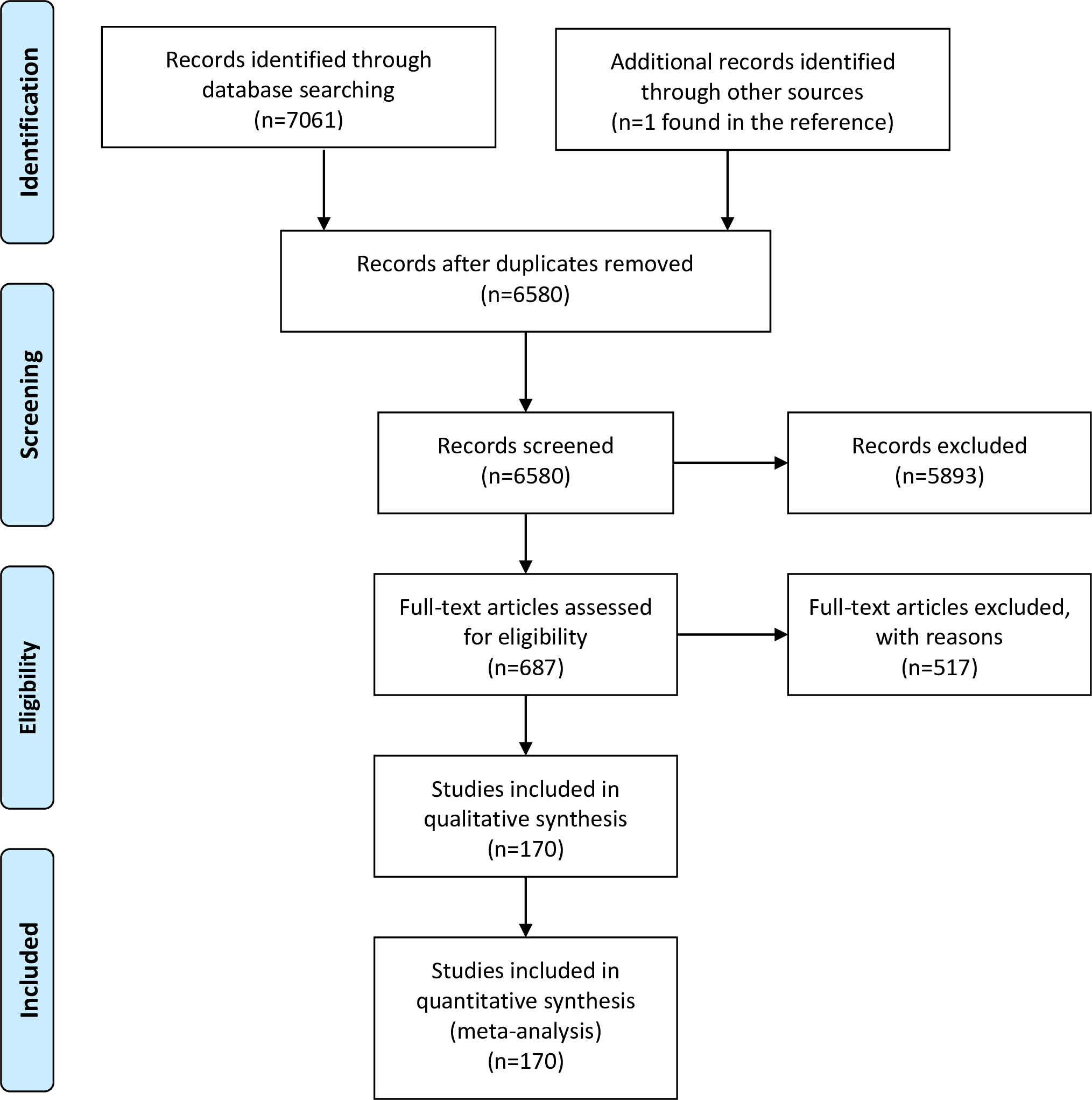

Supplement: S1 Fig — (TIF) [file pmed.1002505.s002.tif]

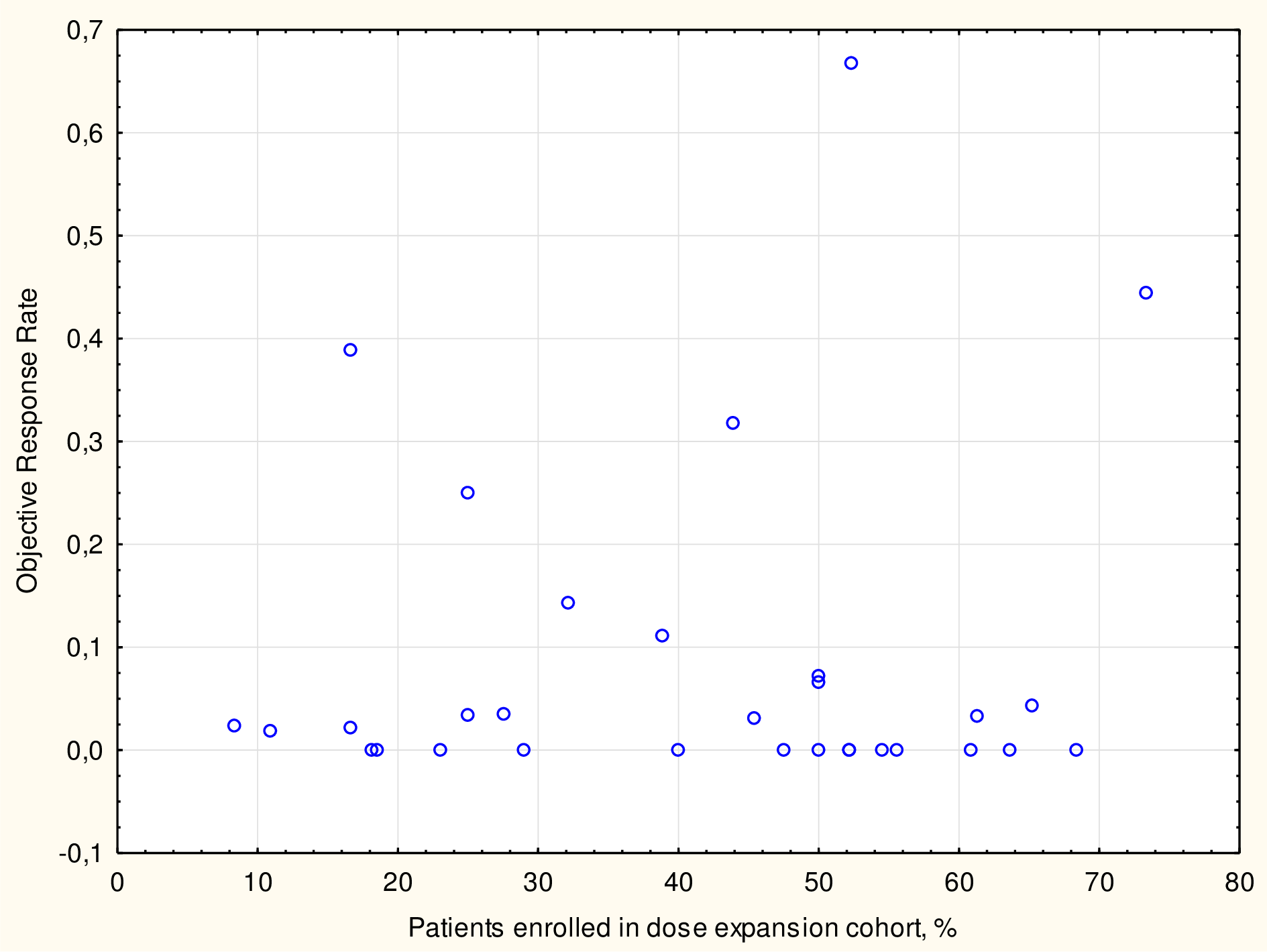

Supplement: S2 Fig — (TIF) [file pmed.1002505.s003.tif]
