# Supplementary material for: Risk and surrogate benefit for pediatric Phase I trials in oncology: A systematic review with meta-analysis
Source: PLoS Med. 2018 Feb 20;15(2):e1002505. doi: 10.1371/journal.pmed.1002505 (PMC5819765; doi:10.1371/journal.pmed.1002505)
Supplement: S1 Table — (DOCX) [file pmed.1002505.s004.docx]

**S1 Table.** Search strategy.

| **EMBASE** |
| --- |
| cancer OR cancers OR carcinoma OR cancerous OR carcinom* OR neoplasm OR neoplas* OR oncology OR oncolog* OR tumor OR tumors OR tumour OR tumours OR 'solid tumor' OR 'solid tumour' OR 'solid tumours' OR malignancy OR malignan* OR leukemia OR leukaemia OR leukemias OR leukaemias OR leucocythaemia OR leucocythemia OR leucocythaemias OR leucocythemias OR (acute AND myeloid AND leukemia) OR (acute AND myeloid AND leukaemia) OR (acute AND lymphoblastic AND leukemia) OR (acute AND lymphoblastic AND leukaemia) OR (acute AND myelogenous AND leukemia) OR (acute AND myelogenous AND leukaemia) OR (hematologic AND malignancy) OR (haematologic AND malignancy) OR (hematologic AND neoplasms) OR (haematologic AND neoplasms) OR (chronic AND myelogenous AND leukemia) OR (chronic AND myelogenous AND leukaemia) OR (juvenile AND myelomonocytic AND leukemia) OR (juvenile AND myelomonocytic AND leukaemia) OR (myelodysplastic AND syndrome) OR (myelodysplastic AND syndromes) OR (transient AND myeloproliferative AND disorder) OR (myeloid AND malignancies) OR (myeloid AND malignancy) OR lymphoma OR (hodgkin AND disease) OR (hodgkins AND disease) OR sarcoma OR osteosarcoma OR (ewing AND sarcoma) OR (ewing AND tumor) OR (ewing AND tumour) OR rhabdomyosarcoma OR (wilms AND tumour) OR (wilms AND tumor) OR nephroblastoma OR retinoblastoma OR medulloblastoma OR teratoma OR germinoma OR (germ AND cell AND tumor) OR (germ AND cell AND tumour) OR seminoma OR gonadoblastoma OR $glioma OR glioblastoma OR astrocytoma OR $carcinoma OR $blastoma AND ('phase 1' AND clinical AND trial OR ('phase 1' AND trial) OR ('phase 1' AND study) OR ('phase 1' AND clinical AND study) OR ('phase i' AND clinical AND trial) OR ('phase i' AND clinical AND study) OR ('phase i' AND study) OR ('phase i' AND trial) OR 'phase 1' OR 'phase i' OR 'phase i and ii' OR 'phase i and phase ii' OR 'phase 1 and 2' OR 'phase 1 and phase 2' OR 'phase 1/2' OR 'phase i/ii' OR ('phase i and ii' AND clinical AND trial) OR ('phase i and ii' AND clinical AND study) OR ('phase 1 and 2' AND clinical AND trial) OR ('phase 1 and 2' AND clinical AND study) OR ('phase 1/2' AND clinical AND study) OR ('phase i/ii' AND clinical AND study) OR ('phase 1/2' AND clinical AND trial) OR ('phase i/ii' AND clinical AND trial) OR ('dose increment' AND study) OR 'dose increment' OR ('dose increment' AND schedules AND study) OR (dose AND increment AND schedules AND study) OR ('dose increment' AND trial) OR ('dose increment' AND schedules AND trial) OR (dose AND increment AND schedules AND trial) OR ('dose escalation' AND study) OR 'dose escalation' OR ('dose escalation' AND schedules AND study) OR (dose AND escalation AND schedules AND study) OR ('dose escalation' AND trial) OR ('dose- escalation' AND schedules AND trial) OR (dose AND escalation AND schedules AND trial) OR ('increasing dose' AND study) OR ('increasing dose' AND trial)) AND (pediatrics OR paediatrics OR paediatric OR pediatric OR children OR child* OR child OR childhood OR adolescent OR infant OR newborn OR toddler OR teen OR 'young adult' OR teenager R preschooler OR 'pre teen') AND [2004- 2015]/py |
| **Search date: 28 Feb 2015 (3514 items)** |
| **PUBMED** |
| ((((cancer OR cancers OR carcinoma OR cancerous OR carcinom* OR neoplasm OR neoplas* OR oncology OR oncolog* OR tumor OR tumors OR tumour OR tumours OR solid tumor OR solid tumours OR solid tumour OR solid tumours OR malignancy OR malignan* OR leukemia OR leukaemia OR leukemias OR leukaemias OR leucocythaemia OR leucocythaemia OR leucocythaemia OR leucocythaemia OR acute myeloid leukemia OR acute myeloid leukaemia OR acute lymphoblastic leukemia OR acute lymphoblastic leukaemia OR acute myelogenous leukemia OR acute myelogenous leukaemia OR hematologic malignancy OR haematologic malignancy OR hematologic neoplasms OR hematologic neoplasms OR chronic myelogenous leukemia OR chronic myelogenous leukaemia OR juvenile myelomonocytic leukemia OR juvenile myelomonocytic leukaemia OR myelodysplastic syndrome OR myelodysplastic syndromes OR transient myeloproliferative disorder OR myeloid malignancies OR myeloid malignancy OR lymphoma OR hodgkin disease OR hodgkins disease OR sarcoma OR osteosarcoma OR ewing sarcoma OR ewing tumor OR ewing tumour OR rhabdomyosarcoma OR wilms tumour OR wilms tumor OR nephroblastoma OR retinoblastoma OR medulloblastoma OR teratoma OR germinoma OR germ cell tumor OR germ cell tumour OR dysgerminoma OR seminoma OR gonadoblastoma OR *glioma OR glioblastoma OR astrocytoma OR *carcinoma OR *blastoma))) AND ((phase 1 clinical trial OR phase 1 trial OR phase 1 study OR phase 1clinical study OR phase i clinical trial OR phase i trial OR phase i'clinical study OR phase i study OR phase 1 OR phase i OR phase i AND ii OR phase i AND phase ii OR phase 1 AND 2 OR phase 1 AND phase 2 OR phase 1/2 OR phase i/ii OR (phase i AND ii clinical trial) OR (phase i AND ii clinical study) OR (phase 1 AND 2 clinical trial) OR phase 1 AND 2 clinical study OR phase 1/2 clinical study OR phase i/ii clinical study OR phase 1/2 clinical trial OR phase i/ii clinical trial OR dose increment study OR dose increment OR dose increment schedules study OR dose increment trial OR dose increment schedules trial OR dose escalation study OR dose escalation OR dose escalation schedule study OR dose escalation trial OR dose escalation schedule trial OR increasing dose study OR increasing dose trial))) AND ((pediatrics OR paediatrics OR paediatric OR pediatric OR children OR child* OR child OR childhood OR adolescent OR infant OR newborn OR toddler OR teen OR young adult OR teenager OR preschooler OR pre-teen)) Filters: Publication date from 2004/01/01 |
| **Search date: 1 Mar 2015 (3547 items)** |
