# Supplementary material for: Risk and surrogate benefit for pediatric Phase I trials in oncology: A systematic review with meta-analysis
Source: PLoS Med. 2018 Feb 20;15(2):e1002505. doi: 10.1371/journal.pmed.1002505 (PMC5819765; doi:10.1371/journal.pmed.1002505)
Supplement: S2 Table — (DOCX) [file pmed.1002505.s005.docx]

**S2 Table.** Inclusion and exclusion criteria.

| **Study Design** |
| --- |
| INCLUSION CRITERIA |
| Pediatric cancer phase 1, defined as "small sample size, non-randomized, dose escalation studies that define the recommended dose for subsequent study of a new drug in each schedule tested." |
| Phase 1 and phase 1/2 (if phase 1 results were reported separately). |
| All types of cancer of any type and stage. |
| EXCLUSION CRITERIA |
| Not a research study (letters to the editors etc.). |
| **Population** |
| INCLUSION CRITERIA |
| Studies where all or most participants were under 21 years old and the study was indicated as pediatric or results were reported separately for pediatric population. |
| Patients with (1) solid tumors, (2) hematological malignancies or 1&2 altogether. |
| EXCLUSION CRITERIA |
| Studies where most participants were 18 years old and/or older. |
| Patients with benign tumor or other disease only, without cancer. |
| Studies with healthy volunteers. |
| **Interventions** |
| INCLUSION CRITERIA |
| Phase 1 and phase 1/2 (if phase 1 results were reported separately) studies where chemotherapy or targeted therapy was the only treatment and drugs were administered systemically: |
| Chemotherapy – cytotoxic drugs schedules monotherapy or polytherapy. |
| Targeted therapy (monoclonal antibodies or small molecules or antibody drug conjugates, in accordance with definitions provided in Karp & Falchook’s Handbook of Targeted Cancer Therapy, 2014) also combinations of targeted therapies. |
| 1&2 altogether. |
| EXCLUSION CRITERIA |
| Studies on drugs administered topically only or regionally only (i.e. directly to the tumor without any systemic effects or minimal systemic effects). |
| Studies where surgery, and radiotherapy were the only treatment. |
| Supportive care without anticancer agents or with other types of drugs and treatments as antiviral agents or non-specific immunotherapy (i.e. Inteferon, interleukins, cytokines, immunostimulator, sagramostim, filgastrim, granulocyte-macrophage colony-stimulating factor [GM-CSF]), cancer vacccine, oncolytic virus therapy). |
| **Outcomes** |
| INCLUSION CRITERIA |
| Drug toxicity and response reported (complete or partial). |
| EXCLUSION CRITERIA |
| Studies reporting only pharmacokinetics and/or pharmacodynamics of a tested treatment. |
