# Supplementary material for: Risk and surrogate benefit for pediatric Phase I trials in oncology: A systematic review with meta-analysis
Source: PLoS Med. 2018 Feb 20;15(2):e1002505. doi: 10.1371/journal.pmed.1002505 (PMC5819765; doi:10.1371/journal.pmed.1002505)
Supplement: S3 Table — (DOCX) [file pmed.1002505.s006.docx]

**S3 Table.** Drugs and drug combinations used in chemotherapy and targeted therapy studies or combinations of chemotherapy and targeted therapy drugs.

| **Chemoterapy** |  | **Targeted therapy** |  | **Combination therapy** |
| --- | --- | --- | --- | --- |
| 5-Fluorouracil (5FU) |  | 17-allylaminogeldanamycin (17-AAG) (2 studies) |  | Bevacizumab, Cyclophosphamide, Sorafenib (2 studies) |
| ABT-751 (2 studies) |  | Aflibercept |  | Bevacizumab, Irinotecan |
| Asparaginase, Clofarabine, Dexamethasone, Etoposide, Mitoxantrone |  | Alisertib (MLN 8237) |  | Bevacizumab, Irinotecan, Temozolomide |
| Azacitidine, Cytarabine, Fludarabine |  | Arsenic trioxide (Trisenox) |  | Bevacizumab, Irinotecan, Temozolomide, Vincristine |
| Bendamustine |  | AT9283 |  | Bortezomib, Dexamethasone, Doxorubicin, PEG-Asparaginase, Vincristine |
| Bleomycin, Cisplatin, Cyclophosphamide, Etoposide |  | Bevacizumab |  | CD19-CAR T, Cyclophosphamide, Fludarabine |
| Carbamazepine, Oxaliplatin |  | Bevacizumab, Temsirolimus |  | Cetuximab, Irinotecan |
| Carboplatin, Ifosfamide, Topotecan |  | BL22 (CAT-3888) |  | Clofarabine, Cytarabine, Sorafenib |
| Carboplatin, Irinotecan |  | Blinatumomab |  | Cyclophosphamide, Doxorubicin, G3139 |
| Carboplatin, Thalidomide |  | Bortezomib (2 studies) |  | Cytarabine + L-Asparaginase, Cytarabine + Mitoxantrone, Gemtuzumab Ozogamicin |
| Carboplatin, Vinblastine |  | Bortezomib (Velcade), Vorinostat (Zolinza) |  | Cytarabine, Doxorubicin, EZN-3042, Methotrexate, Pegaspargase, Prednisone, Vincristine |
| Carmustine (BCNU), O6-benzylguanine (O6-BG) |  | Brentuximab Vedotin (Adcetris) |  | Cytarabine, Etoposide, Quizartinib |
| Cisplatin, Temozolomide |  | Cediranib |  | Erlotinib, Temozolomide |
| Cladribine, Topotecan |  | Ch14.18/CHO |  | Etoposide, Mitoxantrone, Valspodar |
| Clofarabine |  | Cilengitide (EMD 121974) |  | Etoposide, Vorinostat |
| Clofarabine, Cyclophosphamide |  | Cixutumumab |  | Gefitinib, Irinotecan (2 studies) |
| Clofarabine, Cyclophosphamide, Etoposide |  | Cixutumumab, Temsirolimus |  | Irinotecan (IRN), Temsirolimus (TEM), Temozolomide (TMZ) |
| Clofarabine, Cytarabine |  | Combotox (HD37 and RFB4 1.1) |  | Irinotecan, Sorafenib |
| Cyclophosphamide, Decitabine, Dexrazoxane, Doxorubicin |  | Crizotinib |  | Sirolimus, Vinblastine (2 studies) |
| Cyclophosphamide, Etoposide |  | Dabrafenib |  | Temozolomide, Veliparib |
| Cyclophosphamide, Nifurtiomox, Topotecan |  | Dalotuzumab |  | Temozolomide, Vorinostat |
| Cyclophosphamide, Tirapazamine |  | Dasatinib |  |  |
| Cyclophosphamide, Topotecan |  | Depsipeptide |  |  |
| Cyclophosphamide, Vinorelbine |  | Enzastaurin |  |  |
| Cytarabine |  | Erlotinib |  |  |
| Daunorubicin |  | Everolimus |  |  |
| Difluoromethylornithine, Etoposide |  | Fenretinide (2 studies) |  |  |
| Doxorubicin, Oxaliplatin |  | Figitumumab |  |  |
| Ecteinascidin-743 |  | Flavopiridol |  |  |
| Etoposide, Ifosfamide, Oxaliplatin |  | Gemtuzumab ozogamicin (2 studies) |  |  |
| Etoposide, Oxaliplatin (2 studies) |  | Hu14.18-IL2 (EMD273063) |  |  |
| Etoposide, PSC 833 |  | Hu14.18K322A |  |  |
| Etoposide, Temozolomide (2 studies) |  | Imatinib mesylate (STI571) (2 studies) |  |  |
| EZN-2208 (Polyethylene Glycol Conjugate of SN38) |  | Ipilimumab |  |  |
| Fluorouracil, Leucovorin, Oxaliplatin |  | Ispinesib |  |  |
| Gemcitabine |  | Lapatinib |  |  |
| Ifosfamide, Paclitaxel |  | LDE225 (sonidegib, erismodegib) |  |  |
| Ifosfamide, Topotecan |  | LEE011 |  |  |
| Irininotecan (3 studies) |  | Leflunomide (SU101) |  |  |
| Irinotecan, Temozolomide (2 studies) |  | Lenalidomide (2 studies) |  |  |
| Irinotecan, Temozolomide, Vincristine |  | Lestaurtinib |  |  |
| Irinotecan, Topotecan |  | Lexatumumab |  |  |
| Irofulven |  | Lonafarnib |  |  |
| Ixabepilone |  | MK-2206 |  |  |
| Mafosfamide |  | MK0752 |  |  |
| Nelarabine |  | Moxetumomab pasudotox |  |  |
| O6-Benzylguanine, Temozolomide (2 studies) |  | Panobinostat |  |  |
| Oxaliplatin |  | Pazopanib (Votrient) |  |  |
| Paclitaxel |  | Perifosine |  |  |
| Pemetrexed |  | Pracinostat (SB939) |  |  |
| Plitidepsin |  | RG1507 |  |  |
| Raltitrexed |  | Ridaforolimus (MK-8669, AP23573) (2 studies) |  |  |
| Satraplatin |  | Ruxolitinib |  |  |
| Spartaject Busulfan |  | Selumetinib (AZD6244 f) |  |  |
| Temozolomide (2 studies) |  | SGN-CD19A |  |  |
| Temozolomide, Topotecan |  | Sorafenib |  |  |
| Temozolomide, TPI 287 |  | SU5416 (Semaxanib) |  |  |
| Topotecan (3 studies) |  | Sunitinib (Sutent) capsules |  |  |
| Trabectedin |  | Temsirolimus |  |  |
| Vincristine Sulfate Liposomes |  | Temsirolimus, Valproic Acid |  |  |
| Vincristine, Oral Irinotecan and Temozolomide (VOIT), two schedules |  | Tipifarnib |  |  |
| Vinorelbine |  | Tivantinib |  |  |
| VNP40101M Cloretazine |  | Trebananib |  |  |
|  |  | Vandetanib (Caprelsa) |  |  |
|  |  | Vismodegib (Erivedge) |  |  |
|  |  | Vorinostat+/- Retinoic Acid |  |  |
