# Supplementary material for: Risk and surrogate benefit for pediatric Phase I trials in oncology: A systematic review with meta-analysis
Source: PLoS Med. 2018 Feb 20;15(2):e1002505. doi: 10.1371/journal.pmed.1002505 (PMC5819765; doi:10.1371/journal.pmed.1002505)
Supplement: S4 Table — (DOCX) [file pmed.1002505.s007.docx]

**S4 Table.** Objective response rate and toxicity assessed in subgroups.

| **Outcome** | **Type of malignancy** | **Measure** | **Number of drugs** | | **Number of malignancies** | |
| --- | --- | --- | --- | --- | --- | --- |
|  |  |  | **1** | **2 or more** | **3 or less** | **4 or more** |
| **Objective** **responses (122 studies)** | **Solid tumors** | **Number of studies** | 78 | 51 | 17 | 108 |
|  |  | **Response rate (95% CI)** | 2.49%  (1.88-3.11) | 10.54%  (7.61-13.46) | 15.01%  (6.70-23.32) | 2.85%  (2.28-3.42) |
|  |  | ***p*-Value** | <0.001 | | 0.0001 | |
|  | **Hematological malignancies** | **Number of studies** | 24 | 14 | 24 | 14 |
|  |  | **Response rate (95% CI)** | 19.79% (11.47-28.10) | 41.56% (30.13-52.99) | 30.52% (21.37-39.67) | 23.31% (10.75-35.86) |
|  |  | ***p*-Value** | 0.0015 | | 0.35 | |
| **Fatal (grade 5) AEs (43 studies)** | **Solid tumors** | **Number of studies** | 37 | 10 | 10 | 36 |
|  |  | **Grade 5 AE (95% CI)** | 1.79%  (1.02-2.56) | 1.03%  (0.01-2.37) | - | 1.88%  (1.09-2.66) |
|  |  | ***p*-Value** | 0.6726 | | - | |
|  | **Hematological malignancies** | **Number of studies** | 14 | 9 | 16 | 7 |
|  |  | **Grade 5 AE (95% CI)** | 3.74%  (2.20-6.60) | 8.43%  (4.54-12.33) | 4.78%  (2.52-7.04) | 2.33%  (0.02-4.63) |
|  |  | ***p*-Value** | 0.0035 | | 0.0826 | |
| **Grade**  **3/4 AEs (97 studies)** | **Solid tumors** | **Number of studies** | 65 | 38 | 12 | 90 |
|  |  | **Mean number of grade 3/4 AEs per person (95% Cl)** | 0.96  (0.92-1.01) | 2.10  (2.01-2.19) | 0.91  (0.80-1.03) | 1.39  (1.34-1.43) |
|  |  | ***p*-Value** | <0.001 | | <0.01 | |
|  | **Hematological malignancies** | **Number of studies** | 13 | 13 | 19 | 7 |
|  |  | **Mean number of grade 3/4 AEs per person (95% Cl)** | 0.55  (0.48-0.63) | 2.26  (2.08-2.46) | 1.42  (1.31-1.54) | 0.89  (0.78-1.01) |
|  |  | ***p*-Value** | <0.001 | | <0.001 | |

*p*-Value from Q-test for heterogeneity comparing response rate and grade 5 AE rate between type of therapy groups
