# Supplementary material for: Risk and surrogate benefit for pediatric Phase I trials in oncology: A systematic review with meta-analysis
Source: PLoS Med. 2018 Feb 20;15(2):e1002505. doi: 10.1371/journal.pmed.1002505 (PMC5819765; doi:10.1371/journal.pmed.1002505)
Supplement: S5 Table — (DOCX) [file pmed.1002505.s008.docx]

**S5 Table.** Direct comparison of risk and benefit.

| **Outcome** | **Type of malignancy** | **Measure** | **Type of therapy** | | | |
| --- | --- | --- | --- | --- | --- | --- |
|  |  |  | **Chemotherapy** | **Targeted therapy** | **Combined therapy** | **All interventions** |
| **Objective responses in 66 studies in which fatal (grade 5) AEs were reported** | **Solid tumors** | **Number of studies** | 17 | 26 | 2 | 45 |
|  |  | **Response rate**  **(95% CI)** | 10.55%  (2.95-18.15) | 2.32%  (1.29-3.34) | 14.83%  (0.01-31.61) | 2.97%  (2.09-3.84) |
|  |  | ***p*-Value** | 0.0083 | | | - |
|  | **Hematological malignancies** | **Number of studies** | 9 | 8 | 4 | 21 |
|  |  | **Response rate**  **(95% CI)** | 29.33%  (14.68-43.94) | 18.96%  (9.85-28.06) | 38.86% (14.73-62.98) | 26.74%  (18.61-34.88) |
|  |  | ***p*-Value** | 0.0623 | | | <0.001 |
| **Fatal (grade 5) AEs in 66 studies** | **Solid tumors** | **Number of studies** | 17 | 26 | 2 | 45 |
|  |  | **Grade 5 AE rate (95% CI)** | 1.12%  (0.14-2.10) | 1.69%  (0.80-2.58) | 1.68%  (0.10-4.32) | 1.85%  (1.13-2.57) |
|  |  | ***p*-Value** | 0.82 | | | - |
|  | **Hematological malignancies** | **Number of studies** | 9 | 8 | 4 | 21 |
|  |  | **Grade 5 AE rate (95% CI)** | 5.37%  (1.77-8.98) | 3.22%  (0.60-5.83) | 6.33%  (1.15-11.51) | 4.71%  (2.57-6.86) |
|  |  | ***p*-Value** | 0.23 | | | 0.102 |
