# Supplementary material for: Risk and surrogate benefit for pediatric Phase I trials in oncology: A systematic review with meta-analysis
Source: PLoS Med. 2018 Feb 20;15(2):e1002505. doi: 10.1371/journal.pmed.1002505 (PMC5819765; doi:10.1371/journal.pmed.1002505)
Supplement: S6 Table — (DOCX) [file pmed.1002505.s009.docx]

**S6 Table.** Results of previous reviews.

|  | **Type of participants** | **Number of studies** | **Total number of patients** | **Solid tumors** | | **Hematological malignancies** | | **Both types of malignancy** | |
| --- | --- | --- | --- | --- | --- | --- | --- | --- | --- |
|  |  |  |  | **Response rate** | **Drug-related death rate** | **Response rate** | **Drug-related death rate** | **Response rate** | **Drug-related death rate** |
| **Horstmann 2005** | adults | 460 | 11 935 | - | - | - | - | 10.6% | 0.49% |
| **Italiano 2005** | adults | 10 | 180 | 7.2% | 0.5% | - | - | - | - |
| **Kim 2008** | adults | 16 | 262 | 4% | 0.4% | - | - | - | - |
| **Roberts 2004** | adults | 213 | 6 474 | 3.8% | 0.54% | - | - | - | - |
| **Wong 2016** | adults | 49 | 1 353 | - | - | - | - | 2.95% | - |
| **Lee 2005** | pediatric | 69 | 1 973 | - | - | - | - | 9.6% | 0.5% |
| **CURRENT REVIEW** | **pediatric** | **170** | **4604*** | **3.17%**  **(2.62-3.72)** | **1.85%**  **(1.14-2.56)** | **27.90%**  **(20.53-35.27)** | **4.04%**  **(2.18-5.89)** | **10.29%**  **(8.33-12.25)** | **2.09%**  **(1.45-2.72)** |
| * Rates calculated only for a fraction of studies reporting objective response rates and AEs. Please be aware of the differences in methodologies of compared studies. | | | | | | | | |  |
